# A case-control study on factor V Leiden: an independent, gender-dependent risk factor for venous thromboembolism

**DOI:** 10.1186/s12959-021-00328-0

**Published:** 2021-10-19

**Authors:** Vahideh Takhviji, Kazem Zibara, Asma Maleki, Ebrahim Azizi, Sanaz Hommayoun, Mohammadreza Tabatabaei, Seyed Esmaeil Ahmadi, Maral Soleymani, Omid Kiani Ghalesardi, Mina Farokhian, Afshin Davari, Pouria Paridar, Anahita Kalantari, Abbas Khosravi

**Affiliations:** 1 Transfusion Research center, High Institute for Research and Education in Transfusion, Tehran, Iran; 2 PRASE and Biology Department, Faculty of Sciences, Lebanese University, Beirut, Lebanon; 3 Department of hematology, School of Allied Medical Sciences, Tehran University of Medical Sciences, Tehran, Iran; 4 Faculty of Medicine, Ahvaz Jundishapur University of Medical Sciences, Ahvaz, Iran; 5 Department of Hematology and Blood Banking, Faculty of Allied Medicine, Iran University of Medical Sciences, Tehran, Iran; 6 Hematology Department, Tarbiat Modares University, Tehran, Iran; 7 School of Public Health, Tehran University of Medical Sciences, Tehran, Iran; 8 Islamic Azad University, North-Tehran Branch, Tehran, Iran; 9 Department of Anesthesiology, Golestan Hospital, Ahvaz Jundishapur University of Medical Sciences, Ahvaz, Iran

**Keywords:** APCR, Factor V Leiden, Thrombophilia, Venous thromboembolism, DVT, PE, Arterial thrombosis

## Abstract

**Background:**

Activated protein C resistance (APCR) due to factor V Leiden (FVL) mutation (R506Q) is a major risk factor in patients with venous thromboembolism (VTE). The present study investigated the clinical manifestations and the risk of venous thromboembolism regarding multiple clinical, laboratory, and demographic properties in FVL patients.

**Material and methods:**

A retrospective cross-sectional analysis was conducted on a total of 288 FVL patients with VTE according to APCR. In addition, 288 VET control samples, without FVL mutation, were also randomly selected. Demographic information, clinical manifestations, family and treatment history were recorded, and specific tests including t-test, chi-square and uni- and multi-variable regression tests applied.

**Results:**

APCR was found to be 2.3 times significantly more likely in men (OR: 2.1, *p* < 0.05) than women. The risk of deep vein thrombosis (DVT) and pulmonary embolism (PE) in APCR patients was 4.5 and 3.2 times more than the control group, respectively (*p <* 0.05). However, APCR could not be an independent risk factor for arterial thrombosis (AT) and pregnancy complications. Moreover, patients were evaluated for thrombophilia panel tests and showed significantly lower protein C and S than the control group and patients without DVT (*p* < 0.0001).

**Conclusion:**

FVL mutation and APCR abnormality are noticeable risk factors for VTE. Screening strategies for FVL mutation in patients undergoing surgery, oral contraceptive medication, and pregnancy cannot be recommended, but a phenotypic test for activated protein C resistance should be endorsed in patients with VTE.

## Introduction

Thrombophilia, the most common hematologic disorder, is characterized by blood coagulation abnormalities that increases the risk of thrombosis events [1]. Venous Thrombosis (VT) is the third cause of death due to cardiovascular diseases [2]. VT occurs in two general forms known as pulmonary embolism (PE) and deep vein thrombosis (DVT) [3, 4]. Common predisposing factors include aging, surgery, pregnancy, cancer, recent myocardial infarction, hormone therapy in females, prolonged inertia and genetic factors. The latter genetic causes comprise non-O-blood groups, genetic mutations such as G20210A in prothrombin (PTM) gene, deficiencies in protein C (PC), protein S (PS), and antithrombin III (AT-III), as well as activated protein C resistance (APCR) associated with factor V Leiden (FVL) [2, 5–7]. An estimated 64% of patients with venous thromboembolism (VTE) have APCR as the most common associated clotting abnormality [8].

APCR is a hemostatic disorder characterized by the lack of adequate anticoagulant response to activated protein C (APC). More than 95% of cases with APCR abnormality are due to FVL mutation. Dahlback et al. in 1993 reported the first case of APCR created by FVL and in 1994; Bertina and colleagues discovered a single point mutation in the FV gene [7, 9]. APC, along with protein S as a cofactor, degrades Factor Va and Factor VIIIa by cleaving three arginine sites (R306, R506, and R679). The substitution mutation at G1691A results in an amino acid alteration (R506Q), leading to increased thrombin generation and a hypercoagulable state [9, 10]. The risk of thrombosis increase by 5 to 10 fold in patients with heterozygous FVL mutations while homozygotes have an increase of nearly 80 fold [7].

APCR screening tests are based on the anticoagulant activity of APC, and these include modified activated partial thromboplastin time (aPTT), prothrombin time (PT) and snake Russell Viper Venom (RVV) time for patients on heparin and a lupus anticoagulant (LA) [11]. A minimal prolongation of plasma clotting times by exogenous APC in the presence of FVL characterizes the APC-resistant phenotype. In addition, the R506Q mutation genetic defect can be unraveled by DNA techniques [10, 12]. However, the R506Q (FV _Leiden_) is not the only cause of APCR since other mutations such as R306T (FV _Cambridge_), R306G (FV _Hong Kong_), and W1920R (FV _Nera_) can also initiate APCR [13]. Demographics, ethnic, and coagulation factors can influence diagnostic tests and patients’ clinical phenotype. Previous literature suggested a possible correlation between levels of factors V, VIII and IX with APCR parameters [14]. Protein S, protein C and antithrombin III levels may also affect APCR [15, 16]; however, they have not been evaluated before. In these patients, anticoagulants such as heparin, warfarin, or direct oral anticoagulants (DOACs) are used as a treatment. Notably, despite the relatively similar efficiency of all anticoagulants in treating VT, DOACs have shown a lower 2-year risk of VT recurrence after anticoagulant discontinuation [17].

To date, the majority of studies on this disease have been focused on the genotype component. However, clinical manifestations and disease characteristics were not assessed sufficiently, whether independently or in correlation. Given the above background, and due to concerns regarding assay limitations that could adversely affect clinical diagnosis, we aimed to evaluate inherited FV Leiden patients with VTE according to APCR and VET control samples without FV Leiden mutation. The APCR diagnostic and clinical parameters that were studied include the levels of thrombophilia panel, the frequency of adverse thrombotic outcomes, the frequency of adverse pregnancy outcomes associated with FV Leiden, and the influence of gender and age.

## Material and methods

### Study design and patients’ enrollment

This study was designed as a case-control study survey conducted at the Coagulation Center of the Iranian Blood Transfusion Organization (IBTO) in Tehran, Iran. This center is a regional referral center for specialized coagulation testing and rare bleeding disorders. From 2013 to 2018, a total of 288 patients with confirmed FV Leiden abnormality with VTE according to APCR were recruited. Patients with the following criteria were included: 1) thrombotic symptoms (VTE like vein thrombosis and pulmonary embolism), 2) with reduced activity of APC, confirmed by APCR tests, and 3) who have tested for the presence of FV Leiden mutation. Patients excluded from the study were those having other hemostatic bleeding and thrombotic disorders such as an inherited deficiency of protein C, protein S, AT-III or FVIII as well as those who received anticoagulants (warfarin, heparin, etc…), or antiplatelet (aspirin, clopidogrel, etc...) therapies at least 72 h prior to blood sampling. Patients then undertook a detailed history assessment including demographical and clinical characteristics, age at first symptom, patient’s chief complaint, familial history of thrombotic disorders, and their blood groups. In addition, clinical characteristics and laboratory investigations were determined. The control group included a total of 288 VTE patients who were reported negative for FV Leiden after confirmatory tests. This study was approved by the Medical Ethical Committee and the Institute Review Board (IRB) at IBTO. A written consent form was obtained and signed by all study participants for the collection of blood samples.

### Thrombophilia occurrence in the year preceding the inclusion in the study

Before sample collection and in the year preceding the inclusion in the study, the patient’s detailed history was obtained from all participants. This included demographic data, familial history of bleeding and thrombotic disorders, anticoagulants and antiplatelet drugs used, clinical phenotype and thrombotic events (VTE like vein thrombosis and pulmonary embolism), pregnancy complications (such as abortion, eclampsia, preeclampsia, stillbirth, infertility, abortion trimester) and a detailed clinical history of 12 bleeding episodes based on ISTH scoring [18].

### Laboratory work-up

Peripheral venous blood was collected in 3.2% (0.105 M) sodium citrated tubes and centrifuged twice at 2200 x g for 10 min at room temperature to obtain citrated platelet-poor plasma which was then stored at − 80 °C. As a principal challenge, complete assessments were performed in order to classify the patients’ possible disorders and to discriminate between healthy subjects from mild thrombophilic disorders. Among all subjects assessed, those with thrombophilic disorders have been tested for APCR. Standard routine diagnostic assays were performed such as PT, International Normalized Ratio (INR), and aPTT. The presence of LA was confirmed with LA profile and PTT-LA and an abnormal dilute Russell Viper Venom (dRVV) time that demonstrated modification after the addition of the phospholipid rich dRVVT (LA Test and LA Confirm; Gradipore, Australia). Protein C activity was measured by the chromogenic protein C method (Chromogenix, USA) with the normal range being 70 ± 140%. On the other hand, Protein S activity was performed using the Elisa method (protein S normal range is 55 ± 150%). Finally, APC resistance was measured using the APCR test according to the manufacturer’s instructions (Pefakit APC-R FVL, Pentapharm, Basel, CH). To finalize the diagnosis, the levels of Factor VIII, AT-III and fibrinogen were also assessed. FVIII and AT-III were assayed using deficient plasma (Diagnostica Stago, Asnieres, France) whereas Von clauss method was used for Fibrinogen assessment.

### Statistical analysis

Descriptive analyzes were carried out and reported as frequencies and proportions (N, %). Data were analyzed by Kolomogorov-Smirnov test for normality checking. On the other hand, t-test and chi-square tests were used to compare variables in the studied groups (FV Leiden and Control and age and sex subgroups). Then, uni and multivariable ordinal logistic regression were performed to assess the relationship between the risk of FV Leiden and the studied variables. A correlation test was performed to evaluate the relationship between age and the thrombophilia panel. All tests were two-sided with the type I error rate fixed at 0.05 and the significant level for univariable and multivariable analyses assigned 0.25 and 0.05, respectively. Computations were performed using SAS (version 9.4; SAS Institute Inc., Cary, NC, USA) and SPSS for Windows (Version 19) (SPSS Inc., Chicago, IL, USA).

## Results

A total of 288 FVL patients were investigated in this study of whom 189 patients (65.6%) were females and the remaining (99 patients) were males (34.4%) (Table 1). The age of patients ranged from 1 to 80 years old with a mean of 37.87 ± 13.67 years while the control group’s age ranged from 2 to 84 years old with a mean of 33.58 ± 11.8 years. Patients with familial history of thrombosis were ~ 37.7% whereas 66.3% of the patients were new cases with no family history. Demographic data are presented in Table 1.

**Table 1:** Characteristics of studied patients

Variable^a^		
Sex	–	
Female		424 (73.6%)
Male		152 (26.4%)
Age	35.34 ± 12.88	–
Familial History	–	167 (31%)
Manifestations	–	
Bleeding		5 (0.9%)
Thrombophilia		540 (93.8%)
Bleeding and Thrombophilia		16 (2.8%)
Laboratory Work-up		–
PT	14.15 ± 3.7	
APTT	30.46 ± 3.3	
Protein C	117.76 ± 36.6	
Protein S	77.15 ± 22.9	
Anti-Thrombin	100.42 ± 14.3	
APCR	2.57 ± 1.3	
Clinical Manifestation	–	
Arterial Thrombosis		37 (6.4%)
DVT		149 (25.9%)
PE		39 (6.3%)
Abortion		199 (33.3%)
TUS		39 (6.8%)
Abortion Time	–	
First-trim		163 (28.3%)
Second-trim		24 (4.2%)
Third-trim		5 (0.9%)

*PT* prothrombin time, *APTT* activated partial thromboplastin time, *APCR* activated protein C resistance, *AT-III* antithrombin III, *DVT* deep vein thrombosis, *PE* pulmonary embolism, *TUS* thrombosis in unusual sites.

^a^Numerical data are expressed as mean/median and categorical with the percentage

### Demographic characteristics of patients with FV Leiden abnormality

Regarding the presence or absence of FVL, this study contained two groups: APCR and the normal population. There was a significant correlation between gender and APCR abnormality. In fact, despite the significantly higher frequency of women in both groups, the ratio of women in the normal group was particularly (***, *p* < 0.0001) higher than that in the APCR patients (Table 2). Moreover, using the uni- and multivariate logistic regression model, APCR was found to be ~ 2.3 fold significantly higher in men than women, where female sex was a protective variable that remained significant after adjusting for other variables (OR: 2.1, *p* < 0.05) (Table 3).

**Table 2:** Demographic, laboratory, and clinical status of patients with APCR abnormality and normal confirmed final diagnosis. (* *p* < 0.05, ** *p* < 0.01, *** *p* < 0.001)

Variable	Study Population		*P*-value
	APCR	Normal	
Sex			
Female	189 (65.6%)	233 (80%)	
Male	99 (55.6%)	52 (18%)	0.000***
Age (mean ± SD and range)	37 (1–80)	33.5 (2–84)	0.001***
Familial History of Thrombosis	87 (52.1%)	80 (47.9%)	0.18
Type of Disease			
Bleeding	5 (1%)	0 (0.0%)	0.99
Thrombophilia	261 (92.3%)	279 (92.7%)	
Bleeding and Thrombophilia	7 (2.5%)	9 (3.1%)	
Laboratory Findings			
PT	15.32 ± 4.14	13.13 ± 0.74	0.008***
APTT	36.44 ± 9.34	34.63 ± 3.26	0.92
Protein C	94.84 ± 46.58	125.89 ± 28.08	0.000***
Protein S	64.43 ± 29.96	82 ± 20.10	0.000***
Anti-Thrombin	97.58 ± 18.36	98.52 ± 11.64	0.189
APCR	1.47 ± 0.19	3.91 ± 0.78	0.000***
Pregnancy Complications			0.000***
Abortion	65 (22.5%)	127 (44%)	–
Eclampsia	0	0	0.25
Preeclampsia	3 (1%)	2 (0.6%)	0.23
Stillbirth	8 (2.7%)	11 (3.8%)	0.06
Infertility	4 (1.3%)	11 (3.8%)	
Abortion Time			0.17
First Trim	51 (17.7%)	112 (38.8%)	
Second Trim	11 (3.8%)	13 (4.5%)	
Third Trim	3 (1%)	2 (0.6%)	
Thrombotic Complications			
DVT	113 (39.2%)	36 (12.5%)	0.000***
PE	27 (9.3%)	9 (3.1%)	0.001***
Arterial Thrombosis	16 (5%)	21 (7.2%)	0.24

*PT* prothrombin time, *APTT* activated partial thromboplastin time, *APCR* activated protein C resistance, *AT-III* antithrombin III, *DVT* deep vein thrombosis, *PE* pulmonary embolism

**Table 3:** Crude and adjusted differences in APCR diagnosis according to demographic, laboratory, and clinical parameters

Variable	Univariable	Multivariable		
	Adjusted OR (75% CI)	***P-***value	Adjusted OR (95% CI)	***P-***value
Sex				
Female	1	–	–	**–**
Male	2.34	0.000*	**2.13**	0.05**
Age (mean ± SD and range)	1.02	0.001*	1	0.92
Familial History of Thrombosis	1.27	0.18*	1.63	0.24
Laboratory Findings				
PT	1.43	0.001*	0.98	0.94
APTT	3.03	0.08*	0.99	0.97
Protein C	0.97	0.000*	0.99	0.20
Protein S	0.95	0.000*	0.98	0.10
Anti-Thrombin	0.98	0.09*	1	0.70
APCR	0.00	0.95	–	–
Thrombotic Complications				
DVT	4.52	0.000*	**3.53**	0.006**
PE	3.2	0.003*	3.25	0.19
AT-III	0.74	0.39	1.98	0.48
Abortion	0.37	0.000*	0.82	0.67
Abortion Time				
First Trim	–	0.185*		
Second Trim	1.85	0.162*		
Third Trim	3.29	0.199*		

*PT* prothrombin time, *APTT* activated partial thromboplastin time, *APCR* activated protein C resistance, *AT-III* antithrombin III, *DVT* deep vein thrombosis, *PE* pulmonary embolism

The age of patients ranged from 1 to 80 years (median 37); however, 58.6% of patients were in the age interval of 20 to 50 years, whereas 20% were between 50 to 60 years. Regarding the autosomal recessive inheritance of this disease, the familial history was evaluated in APCR patients and controls. Indeed, the APCR abnormality was 1.2 times more likely to occur in individuals with family history (Odds ratio of 1.27, and a 75% CI of 0.63 to 0.87) (Table 3). As shown in this table, sex, young age, and familial history of thrombosis were predictive factors for FV Leiden.

### Laboratory assessments

Results showed significant differences in laboratory findings between patients and controls. Overall, most APCR patients showed normal PT and aPTT with; respectively, only 10.8% and 5.6% of patients harboring abnormal interpretations. Similarly, all normal controls also revealed normal PT and aPTT results; however, their mean values were significantly lower than those in the APCR group (Table 2). Therefore, we measured the association of PT and PTT results with APCR abnormality by uni- and multivariable regression model. Increased PT in the APCR group was 1.43 fold more likely than the control group, which remained significant after adjustment for other variables in a multivariate analysis (Table 3). In addition, the risk of APCR diagnosis was increased three-fold in prolonged PTT situations (Table 3). Patients were then evaluated for thrombophilia panel tests; protein C¸ protein S and anti-thrombin. Interestingly, results showed that FV Leiden patients had significantly lower PC and PS levels than the control group (***, *p* < 0.0001), however, without a significant difference in anti-thrombin levels (Table 2). Using the logistic regression model, the thrombophilia panel was also identified as a highly valuable test (***, *p <* 0.0001) in univariate analysis (Table 3). Moreover, APCR patients experiencing DVT episodes had significantly lower levels of PC (*p* = 0.003), PS (*p* = 0.000) and, anti-thrombin III (p = 0.007) than patients without DVT. On the other hand, all thrombophilia tests including PC, PS, and anti-thrombin had a strong negative correlation with PT and aPTT results in both groups that were statistically significant in APCR patients only (data not shown). FVL patients also exhibited significantly lower levels of APCRPenta, used as a confirming test.

### Thrombophilia panel and screening tests according to age and gender

Since APCR abnormality revealed a varied pattern in the age of manifestations and sex subgroups, compared to the normal group, therefore, changes in laboratory tests and thrombophilia panel were evaluated at different ages and sex subtypes. Thrombophilia panel in APCR patients showed a negative correlation with age, however, this did not reach statistical significance. There was no significant correlation between screening tests and sex subgroups except PrC, for which females showed a significantly higher mean rank range than males (*P* = 0.000).

### Thrombotic clinical manifestations

Patients were divided into 3 groups according to their type of symptoms: bleeding disorder, thrombophilia, and bleeding and thrombophilia. More than 94% of subjects were thrombophilic and about 3% were thrombophilic-hemorrhagic. In the APCR group, 5 patients had just bleeding symptoms such as menorrhagia, coetaneous bleeding, epistaxis, GI bleeding, bleedings after tooth extraction and surgery. In fact, one patient had a total score of 21, three patients with a score of 3, and one patient with a score of 2.

**Fig. 1:**
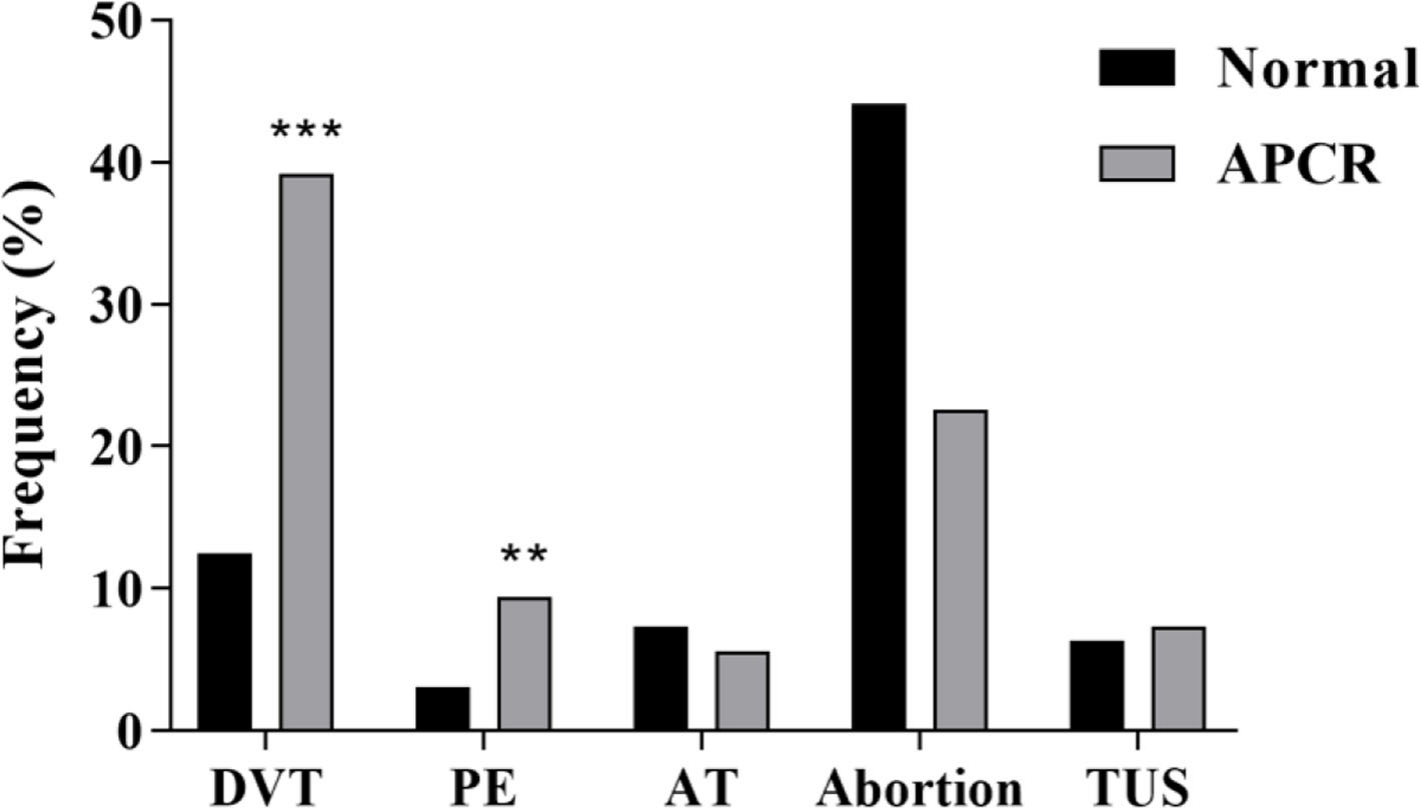
Clinical manifestations of confirmed APCR patients compared to the control group in the year preceding inclusion in the study. (DVT: Deep Vein Thrombosis, PE: Pulmonary Embolism, AT: Arterial Thrombosis, TUS: Thrombosis in Unusual Sites) (* *p* < 0.05, ** *p* < 0.01, *** *p* < 0.001)

The frequency of each clinical symptom was assessed and compared between the two groups. The most common manifestations in patients with APCR abnormality were DVT (39.2%) followed by abortion (22.6%) and PE (9.4%) (Fig. 1). About 75% of DVT and PE were seen in APCR patients and the rest of them in normal controls. The frequency of DVT and PE in the APCR group was significantly higher than that in the control group. Moreover, using the uni- and multivariate logistic regression model, the risk of DVT and PE in APCR patients, was 4.5 and 3.2 times higher than the normal group, respectively. This risk remained significant after adjusting for other variables (Table 3).

Abortion, the second most common symptom in APCR patients, was the most common symptom in normal patients who showed ~ 66% of all abortions. Abortion in both groups occurred most frequently in the first trimester of pregnancy and the two groups did not show a significant difference in abortion trimester (Tables 2 and 3). Other pregnancy complications such as eclampsia, pre-eclampsia, stillbirth, and infertility were also assessed and there was no significant difference between the two groups (Table 2).

**Fig. 2:**
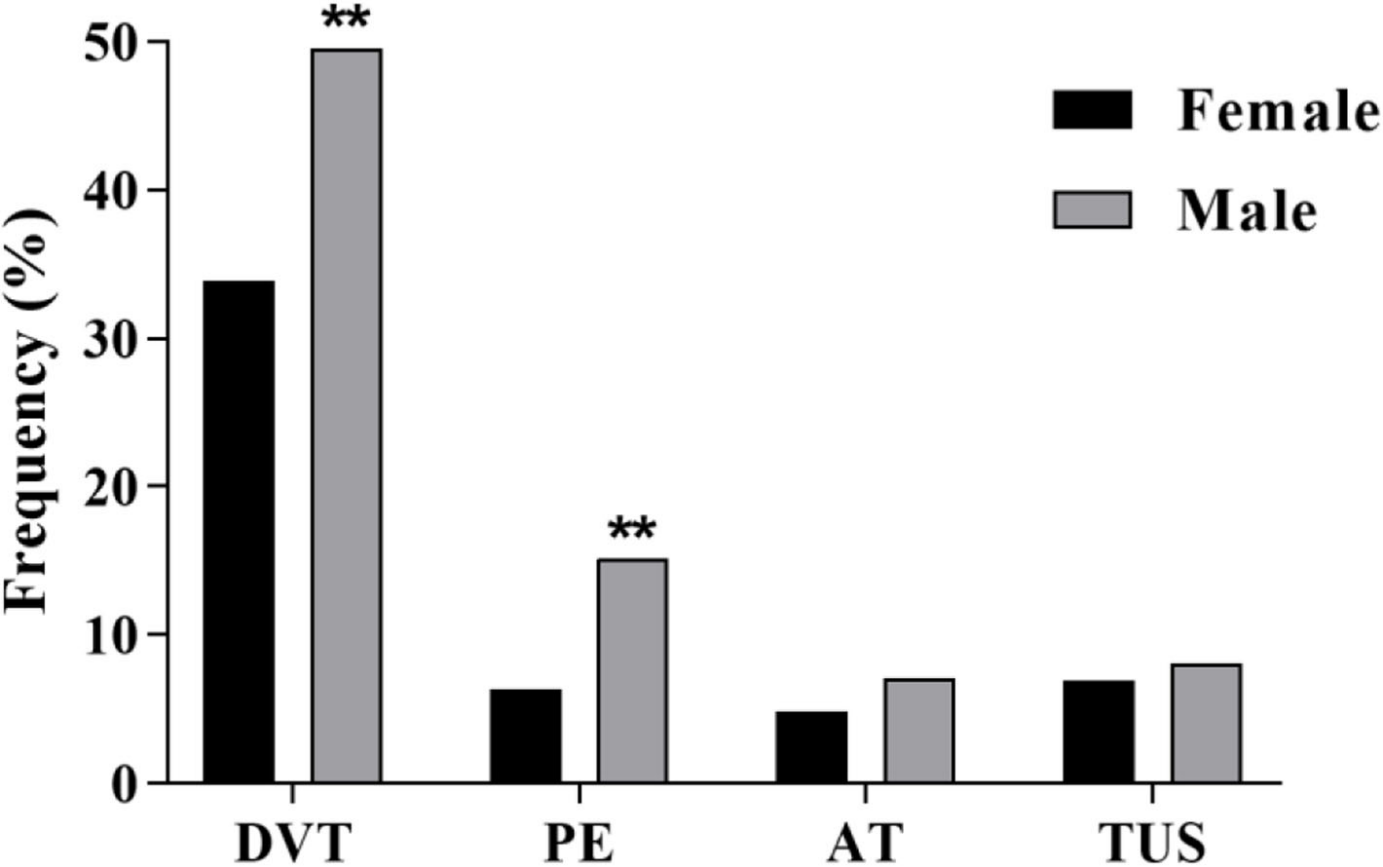
Comparison of thrombotic symptoms among males and females in APCR patients. (DVT: Deep Vein Thrombosis, PE: Pulmonary Embolism, AT: Arterial Thrombosis, TUS: Thrombosis in Unusual Sites) (* *p* < 0.05, ** *p* < 0.01, *** *p* < 0.001)

We then investigated separately all thrombotic symptoms in different demographic groups. Chi-Square test results revealed significant correlations between DVT and PE symptoms with sex subgroups. Despite the higher frequency of women, the incidence of DVT and PE symptoms was significantly higher in men (*p* = 0.01) (Fig. 2).

**Fig. 3:**
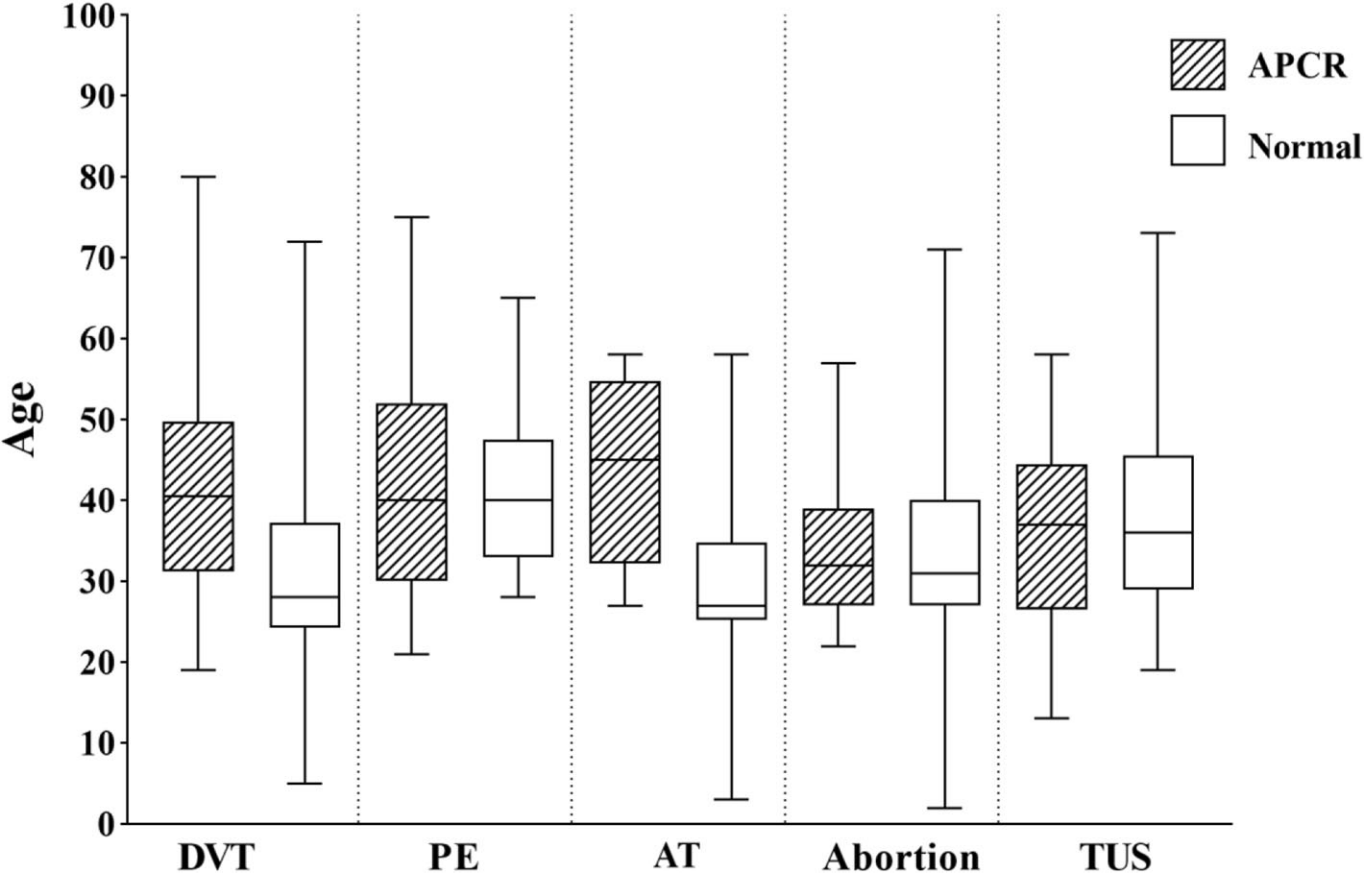
The pattern of thrombotic symptoms in APCR patients and control groups of different ages. (DVT: Deep Vein Thrombosis, PE: Pulmonary Embolism, AT: Arterial Thrombosis, TUS: Thrombosis in Unusual Sites)

Finally, the incidence of symptoms at age intervals was assessed in both normal and APCR groups. All symptoms in APCR patients occurred at a young age. The inter-quartile range or the mean age dispersion of symptoms was low for FV Leiden patients and ranged between 30 to 50 years for all symptoms. Results showed that individuals with Arterial Thrombosis (AT) and DVT in APCR patients had a mean age higher than normal controls (Fig. 3).

## Discussion

APCR is the most common outcome in individuals with familial thrombophilia, as patients have a heightened risk of developing thrombosis [1]. The inheritance of this abnormality is autosomal recessive and its frequency varies between countries. The prevalence of FVL in some parts of the world, such as Japan and Africa, is null; however, it is 5–10% in Europe. In Tehran, a province with different nations in Iran, the prevalence was reported to be ~ 5.5% [7, 12, 19, 20]. Consanguineous marriages in Iranian culture play an important role in the development of hereditary disorders [21]. Therefore, we focused in this study on the demographic¸ clinical and laboratory characteristics as well as on estimating the risk of venous thromboembolism in patients showing resistance to APC in the Iranian population.

APCR abnormality is associated not only with genetic mutations but also with a number of factors including sex, age, anticoagulant, and antiplatelet agents. Previous studies have shown that APCR along with the female gender, appeared to increase the risk of thrombosis and that the mean APC ratio is significantly lower in females than in males [22]. Interestingly, in the present study, despite the higher frequency of women in both APCR and control groups, more than 65% of men were resistant to APC which was found to be significantly more likely in men than women, in contradiction to other previous reports [23, 24]. Indeed, although twice as many females than males were included in the study; however, we found a gender-related difference for APCR which was more likely to develop in men. This could reflect the fact that women are generally more investigated than men for inherited thrombophilia [25]. We suggest that a comprehensive study should be performed on the gender-related differences of APCR abnormality. On the other hand, the onset of thrombosis in the APCR group occurred at a young age, with a narrower range of 20–50 years, than the control group. Our data is in accordance with previous studies which showed that APCR is a significant thrombosis cause in younger individuals [26, 27].

Here, we describe the association of resistance to APC with VTE. Participants bearing APCR abnormality had a clearly significant increased risk for VTE. The age and sex-adjusted incidence odds ratio for the first episode of VTE was 3.53, consistent with the data reported by Ridker and Francesco [28, 29]. The most common manifestations in patients with APCR abnormality were DVT followed by abortion and PE. Other reported symptoms were AT and TUS. Despite the higher frequency of women, the second thrombotic symptom was miscarriage (22%) along with pregnancy complications (5%), accounting together for 27% of all symptoms. This percentage in the APCR group was significantly lower than that in normal women controls which harbored more than 66% of all abortions. Previous studies reported that APC-resistant women experience their first thrombotic event at fertility, associated with both oral contraceptive use and pregnancy [30–32]. This may in part explain that one of the relevant risk factors associated with the first event are oral contraceptive medication, pregnancy, and postpartum because women also had a lower average age than men [32]. However, given that abortions and pregnancy complications were higher in normal controls, FVL cannot be considered independently as the cause of these symptoms in APCR patients, but this is rather multifactorial.

In APC-resistant patients, only 11.8 and 9% of total events were AT and PE. The association of AT with resistance to APC is a controversial issue. In our study, the mean age of patients with AT was higher than those with other symptoms and also higher than the control group. In accordance, a previous study revealed that AT is associated with other risk factors at an advanced age [33]. All thrombotic symptoms were also compared in sex subgroups when gynecological symptoms were omitted. As mentioned above, despite the significantly higher frequency of women investigated, APCR was found significantly more likely in men. Moreover, thrombotic complications including DVT, PE, and AT were significantly higher in men than women. This could indicate a clinically relevant gender difference and reflect an increased DVT and PE risk in men, highlighting the low risk of VTE caused by oral contraceptive use in female carriers [34].

## Conclusion

In conclusion, patients with phenotypic resistance to APC have an increased risk for VTE. This study suggests that women experience their first thrombotic event at a younger age; however, factor V Leiden cannot be independently the cause of abortion and pregnancy complications in APCR patients. Finally, thrombotic events are found to be significantly more likely in men. Screening strategies for factor V Leiden mutation in patients undergoing surgery, oral contraceptive medication, and pregnancy cannot be recommended, but a phenotypic test for activated protein C resistance should be endorsed in patients with VTE.

### Limitations

The size of studied population and some incomplete questionnaires was the limitations of this study.

## Data Availability

The datasets used and/or analyzed during the current study are available from the corresponding author on reasonable request.

## Abbreviations

VT: Venous Thrombosis
PE: Pulmonary embolism
DVT: Deep vein thrombosis
PTM: Prothrombin
PC: Protein C
PS: Protein S
AT-III: Antithrombin III
FVL: Factor V Leiden
APC: Activated protein C
aPTT: Activated Partial Thromboplastin Time
PT: Prothrombin time
RVV: Russell Viper Venom
LA: Lupus anticoagulant
IBTO: Iranian Blood Transfusion Organization
IRB: Medical Ethical Committee and the Institute Review Board
INR: International Normalized Ratio
dRVV: Dilute Russell Viper Venom
